# Accuracy of Emergency Physician-Performed Echocardiography for Diastolic Dysfunction in Suspected Acute Heart Failure: A Systematic Review and Meta-Analysis

**DOI:** 10.3390/jcm14217726

**Published:** 2025-10-30

**Authors:** Shao-Min Huang, Yu-Hsuan Yeh, Cheuk-Kwan Sun, Hsuan-Wei Chu

**Affiliations:** 1 Department of Medical Education, Show Chwan Memorial Hospital, Changhua 500, Taiwan; jamesxxx1997@gmail.com; 2 Department of Medical Education, Cheng Ching Hospital, Taichung 407, Taiwan; yehyeh86122@gmail.com; 3 Department of Emergency Medicine, E-Da Dachang Hospital, I-Shou University, Kaohsiung 807, Taiwan; 4 School of Medicine for International Students, I-Shou University, Kaohsiung 840, Taiwan; 5 Division of Cardiology, Department of Internal Medicine, Chung Shan Medical University Hospital, Taichung 402, Taiwan; 6 Institute of Medicine, Chung Shan Medical University, Taichung 402, Taiwan

**Keywords:** diastolic dysfunction, heart failure with preserved ejection fraction, focused cardiac ultrasound, dyspnea, emergency department

## Abstract

**Background**: This study compared the accuracy of diagnosing left ventricular diastolic dysfunction (DD) between emergency physician (EP)-performed focused cardiac ultrasound (FCU) and cardiologist-interpreted reference standards in adult patients presenting to the emergency department (ED) with suspected acute heart failure (HF). **Methods**: PubMed, Embase, and the Cochrane Central Register of Controlled Trials were systematically searched for studies focusing on adult ED patients with symptoms of acute HF that compared the accuracy of DD diagnosis between EP-performed FCU and reference standards including cardiologist-reviewed FCU clips, formal transthoracic echocardiography, or invasive hemodynamics without language/date restrictions. **Results**: Meta-analysis of four eligible observational studies (327 patients enrolled; 298 analyzed) demonstrate excellent sensitivity of EP-performed FCU compared with the standards (94%, 95% CI: 87 to 97%) but moderate specificity (59%, 95% CI: 42 to 74%) in diagnosing DD patients with suspected acute HF. Three of the four studies showed a high risk of overall bias. **Conclusions**: EP-performed FCU exhibited excellent sensitivity and low negative likelihood ratios, supporting its use as an effective initial triage tool for ruling out DD in ED patients with suspected acute HF. However, its moderate specificity and limited positive likelihood ratios hamper its standalone use as a definitive diagnostic modality for ruling in DD (PROSPERO: CRD420251046794).

## 1. Introduction

Left ventricular diastolic dysfunction is a critical contributor to acute heart failure regardless of ejection fraction (EF) [1]. Diastolic dysfunction involves increased stiffness of the left ventricle with compensatory elevation in filling pressures and a reduced stroke volume, thereby triggering heart failure symptoms particularly during exertion even when the EF is preserved [2,3,4,5,6]. Heart failure with preserved EF (HFpEF), which involves elevated left-ventricular filling pressures, comprises approximately half of all heart failure population [7]. Notably, one in six patients over 65 with unexplained exertional dyspnea has unrecognized heart failure, mostly HFpEF, underscoring diastolic dysfunction-driven HFpEF as a frequent yet underdiagnosed cause of acute breathlessness [8,9].

Despite being the reference-standard modality for assessing diastolic function, formal TTE (trans-thoracic echocardiography) is seldom immediately available as a point-of-care diagnostic tool in the emergency department (ED). To bridge the diagnostic gap, emergency physicians increasingly utilize focused cardiac ultrasound, incorporating simplified Doppler indices and supportive markers such as left atrial size for prompt evaluation of diastolic function [10,11,12,13,14].

However, assessing diastolic dysfunction via focused cardiac ultrasound (FCU) in the ED remains technically challenging. Major international guidelines caution against relying solely on FCU for diastolic evaluation. Consensus statements from the American Society of Echocardiography/American College of Emergency Physicians (ASE/ACEP, 2010) and the European Society of Cardiology’s Acute Cardiac Care group (ESC/EACVI, 2013) uniformly recommend formal TTE over FCU without advanced training for accurate diastolic assessment [15,16]. On the other hand, with accumulating studies aiming at validating emergency physician-performed diastolic function assessment against formal TTE [10,11,12], the 2023 Advanced Emergency Medicine Ultrasonography (AEMUS) Core Content revision, led by the American Board of Emergency Medicine (ABEM), explicitly lists diastolic function assessment as a core advanced cardiac ultrasound competency [17]. Several single-center ED studies have explored the reliability of adopting simplified FCU approaches for assessing diastolic dysfunction in patients with suspected acute heart failure and reported promising diagnostic performance [10,11,12,13,14]. Nevertheless, to date, the results of those studies remain disparate, possibly attributable to a lack of formal synthesis and a standardized diagnostic pathway.

Therefore, this meta-analysis compared the reliability and clinical applicability of emergency physician-performed focused cardiac ultrasound (EP-FCU) with cardiologist-interpreted reference standards in identifying diastolic dysfunction regardless of the ejection fraction.

In accordance with the PIT format (i.e., Population/Index test/Target condition) based on the Cochrane Handbook for Systematic Reviews of Diagnostic Test Accuracy (Cochrane DTA) [18], the current systematic review aimed at updating the current evidence regarding the diagnostic accuracy of EP-FCU (Index test) among adults presenting to the emergency department with symptoms suggestive of acute heart failure (Population) compared with that of formal TTE, cardiologist-reviewed ultrasound clips, or invasive hemodynamic measurements that served as reference tests for identifying the presence of left ventricular diastolic dysfunction (Target Condition).

## 2. Materials and Methods

The current meta-analysis and systematic review followed the Preferred Reporting Items for Systematic Reviews and Meta-Analyses of Diagnostic Test Accuracy (PRISMA-DTA) guidelines (Supplementary Files S1 and S2) [19] and adhered to the methodological recommendations outlined in the Cochrane Handbook for Diagnostic Test Accuracy Reviews [18] for study selection, review, and evidence synthesis. This study was prospectively registered with PROSPERO (registration ID: CRD420251046794).

### 2.1. Source and Search Strategy

Two independent reviewers (SMH and YHY) systematically searched electronic databases including PubMed (1946–present), Embase (Elsevier, 1947–present), and the Cochrane Central Register of Controlled Trials (CENTRAL) on 6 May 2025. No restrictions regarding language, publication date, or study type were imposed. Potentially eligible articles were identified by combining controlled vocabulary (MeSH/Emtree) and free-text terms for the index test (i.e., “focused cardiac ultrasound”, “point-of-care ultrasound”, “tissue Doppler”, “E/e′”), the target condition (i.e., “diastolic dysfunction”, “heart failure with preserved ejection fraction”), and the emergency care setting/personnel (i.e., “emergency department”, “emergency physician”) using Boolean operators and database-specific syntax. Reference lists of identified studies and relevant review articles were manually screened to identify additional eligible studies. The detailed search strategies and full list of search terms are provided in Supplementary File S3.

### 2.2. Eligibility Criteria and Study Selection

Studies were eligible for inclusion if they recruited adult patients (≥18 years) presenting to the emergency department with acute dyspnea or other clinical symptoms suggestive of acute heart failure. The index test of interest was EP-FCU, utilizing any view or Doppler parameter specifically for assessing diastolic function. Acceptable reference standards included either (a) FCU clips reviewed by a cardiologist or accredited sonographer, (b) formal TTE, or (c) invasive hemodynamic assessment performed during the same clinical encounter.

We considered prospective or retrospective observational diagnostic accuracy studies without language or publication date restrictions. Conference abstracts were eligible if they reported adequate data to construct a 2 × 2 contingency table. Studies enrolling fewer than 20 participants were excluded. All retrieved publications from electronic database searches were imported into EndNote 21 (Clarivate, Denver, CO, USA) for Mac to remove duplicates. The remaining records were then exported into the web-based software Rayyan (web-based application without public version) [20] for the initial title and abstract screening.

### 2.3. Screening and Assessment of Studies

The same two independent reviewers (SMH and YHY) screened the retrieved records based on titles and abstracts and subsequently reviewed full-text articles against predefined inclusion criteria. Disagreements were resolved through discussion with a third reviewer (HWC) till a consensus was reached. During the initial screening phase, clearly irrelevant records were excluded based on predetermined exclusion criteria. Full-text articles for studies that passed initial screening were then retrieved and rigorously assessed for eligibility against the predefined inclusion and exclusion criteria. In addition, the reference lists of the included articles were manually examined to identify any potentially relevant studies not captured by the original database searches. Reasons for exclusion of individual studies are documented in Supplementary File S4.

### 2.4. Data Extraction

Essential data from eligible papers were independently extracted by the same two reviewers (SMH and YHY) to a bespoke data extraction form that included information regarding study characteristics (e.g., author, year of publication, country) and study details (e.g., sample size, patient demographics, index-test protocol, reference-standard criteria). Primary outcomes included pooled sensitivity and specificity, while secondary outcomes comprised positive likelihood ratio (PLR) and negative likelihood ratio (NLR).

We dichotomized diastolic dysfunction as positive for any grade (I–III) and negative for normal findings. When a study reported an indeterminate/uninterpretable category for either the index test (EP-FCU) or the reference standard, we excluded such cases from 2 × 2 extraction while recording the per-study counts. This rule was applied consistently across studies.

### 2.5. Risk of Bias Assessment

The same two independent reviewers (SMH and YHY) assessed the risk of bias for the included studies using the Quality Assessment of Diagnostic Accuracy Studies—2 (QUADAS-2) tool [21], specifically tailored to this systematic review. Reviewers were blinded to each other’s ratings during this process.

The QUADAS-2 tool evaluates four primary domains relevant to diagnostic accuracy studies: patient selection, index test (emergency physician-performed assessment of diastolic dysfunction), reference standard (stored FCU clips reviewed by a cardiologist or accredited sonographer, formal TTE, or invasive hemodynamic measurement), and the flow and timing of the study processes. Each domain was categorized as having a low, high, or unclear risk of bias. A summary plot illustrating the overall assessment of risk of bias across included studies was generated using the Robvis visualization tool (Shiny web app; no public version) [22].

### 2.6. Statistical Analysis and Data Synthesis

Indeterminate (or “uninterpretable”) results were defined a priori as initially enrolled participants in whom either the index test (EP-performed focused cardiac ultrasound, EP-FCU) or the reference standard could not yield a definitive classification of diastolic function. We constructed study-level 2 × 2 tables using a complete-case rule: Only participants with a conclusive “positive” (the presence of DD regardless of grading) or “negative” (normal) classification on both the index test and the reference standard contributed to the primary meta-analysis, while those labelled indeterminate by either test were excluded from the 2 × 2 tables. The numbers of patients with definitive classifications and those who were excluded due to indeterminate results were recorded and reported.

All statistical analyses were conducted using Stata 18 with the metandi command. A bivariate random-effects model was adopted to derive pooled estimates of sensitivity and specificity, along with corresponding 95% confidence intervals (CIs), presented via coupled forest plots. Moreover, we generated a hierarchical summary receiver-operating characteristic (HSROC) curve and summary point to assess the overall diagnostic performance [23].

We prespecified sensitivity analysis using Meta-DiSc 2.0 [24] that excluded the small, high-risk-of-bias studies and reported pooled sensitivity, specificity, PLR, and NLR for the sensitivity analysis.

## 3. Results

### 3.1. Search Results

**Figure 1:**
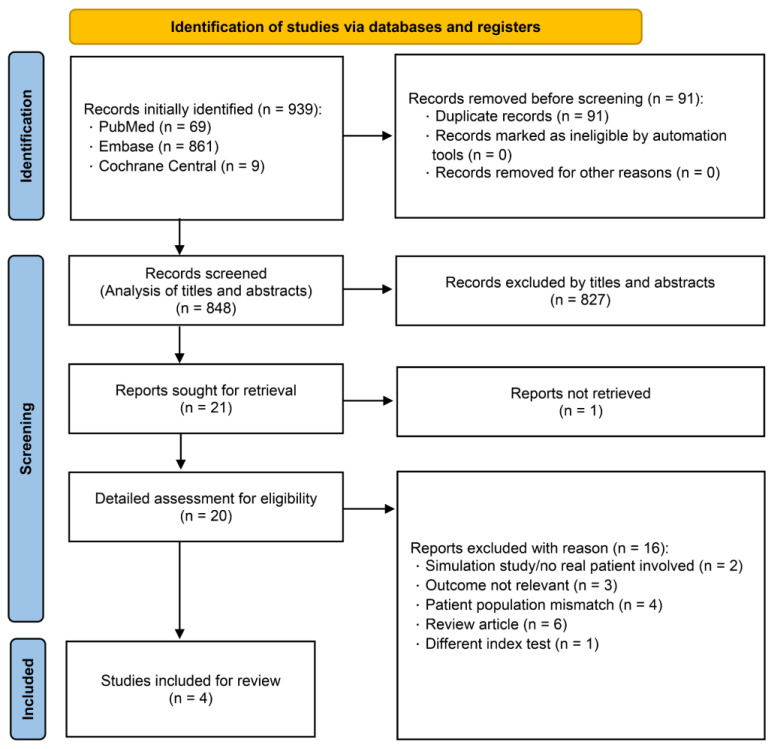
PRISMA-DTA flow diagram.

Of a total of 939 studies initially retrieved through our systematic search, 20 deemed potentially eligible following title and abstract screening were subjected to a full-text review. Eventually, four prospective observational studies met all predefined inclusion criteria. The detailed selection process is depicted in Figure 1. Of the 327 patients initially enrolled in the four eligible studies, 298 eventually contributed to our pooled analysis after excluding those with indeterminate classifications. In detail, 69 patients were included with no indeterminate case in the study by Ünlüer et al. [10], 44 were recruited after excluding 18 indeterminate cases from 62 patients initially enrolled in the study by Ehrman et al. [11], 20 were included with no indeterminate case in the study by Moeller et al. [25], and 165 were enrolled following the exclusion of 11 indeterminate cases from 176 patients initially recruited in the study by Lin et al. [12].

### 3.2. Description of Included Studies

**Figure 2:**
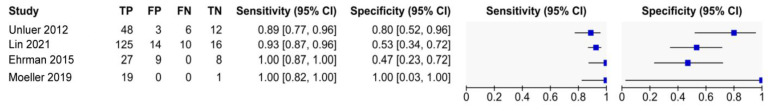
Forest plot comparing the sensitivity and specificity of emergency physician-performed focused cardiac ultrasound with ultrasound interpretation by cardiologists. Data were extracted from studies by Ünlüer et al., 2012 [10]; Lin et al., 2021 [12]; Ehrman et al., 2015 [11]; and Moeller et al., 2019 [25]. TP = true positive; FP = false positive; FN = false negative; TN = true negative. (PS: Of the 62 and 176 patients initially recruited in the studies by Ehrman et al. and Lin et al., 18 and 11 were excluded, respectively, giving sample sizes of 44 and 165 as analyzable denominators for determining pooled estimates).

The characteristics of the four eligible studies are shown in Table 1, while the diagnostic performance of EP-FCU for diastolic dysfunction is summarized in Table 2. The forest plot of the four studies, compiled using Review Manager 5.4.1 [26], is displayed in Figure 2. A comprehensive list of all excluded studies with specific reasons for their exclusion is available in Supplementary File S4.

In the prospective observational study by Ünlüer et al. (2012) [10], three attending emergency physicians received a brief diastology training course (i.e., three-hour didactic session and three-hour hands-on practice) before performing limited bedside FCU on 69 dyspneic adults (mean age 63 ± 9 years; all EF ≥ 50%). They assessed basic trans-mitral Doppler indices (E/A ratio, deceleration time, isovolumic relaxation time), which were subsequently compared with those acquired with formal TTE performed by a cardiologist within two hours using tissue Doppler-based diagnosis. Stringent exclusion criteria of that study (age < 40 years, pregnancy, acute coronary syndrome, atrial fibrillation, significant valvular disease, or tachycardia) may have optimized imaging conditions, potentially enhancing diagnostic accuracy. That study demonstrated a sensitivity of 89% (95% CI, 77 to 96%) and specificity of 80% (95% CI, 52 to 96%) of EP-FCU, suggesting reasonable diagnostic utility.

In the study conducted by Ehrman et al. (2015) [11], two ultrasound fellowship-trained emergency physicians underwent identical diastology training (i.e., three-hour didactic plus three-hour hands-on) and performed FCU on 62 adults (mean age 56 ± 14 years; 48% male) presenting with undifferentiated dyspnea. They employed both trans-mitral Doppler (E/A ratio) and tissue Doppler parameters (i.e., septal & lateral e′, E/e′), according to simplified 2009 American Society of Echocardiography (ASE) criteria. The reference standard was a blinded cardiologist’s reinterpretation of emergency physician acquired images. Diagnostic performance indicated high sensitivity (100%, 95% CI: 87 to 100%) but limited specificity (47%, 95% CI: 23 to 72%), reflecting notable limitations in positive predictive utility.

In the small-sample study by Moeller et al. (2019) [25], FCU was performed by an ultrasound fellowship-trained emergency physician using pulse-wave and tissue Doppler measurements with the findings compared to formal sonography interpreted by a blinded cardiologist. Among the 20 patients evaluated, the emergency physician correctly identified 19 patients as positive for diastolic dysfunction and one patient as negative based on the cardiologist’s assessment. This resulted in high sensitivity (i.e., 100%, 95% CI: 82 to 100%) and high specificity (i.e., 100%, 95% CI: 3 to 100%).

Lin et al. (2021) [12] further expanded these findings in a study involving twelve ultrasound-fellowship-trained emergency physicians, who completed a comparable diastology training program (i.e., two-hour didactic plus two-hour hands-on). They performed a comprehensive FCU evaluation (including E/A ratio, septal e′, lateral e′, E/e′, and left atrial size) on 176 patients (mean age 64 ± 14 years; 65% male; 63% EF < 50%) presenting with dyspnea, syncope, or chest pain. Their scans were validated against same-day cardiologist-performed TTE using a modified 2016 ASE diastolic algorithm with a septal e′ cutoff set at <8 cm/s and an average interval of approximately four hours between scans. EP-FCU exhibited high sensitivity (93%, 95% CI: 87 to 96%) but moderate specificity (53%, 95% CI: 34 to 72%), highlighting similar diagnostic characteristics to those reported by Ehrman et al.

### 3.3. Overall Diagnostic Values of EP-FCU for Diastolic Dysfunction

Across all four studies, EP-FCU demonstrated high sensitivity (89 to 100%) and an excellent NLR (0 to 0.14) but moderate specificity (47 to 100%) and a modest PLR (1.89 to 4.44) for diastolic dysfunction.

**Figure 3:**
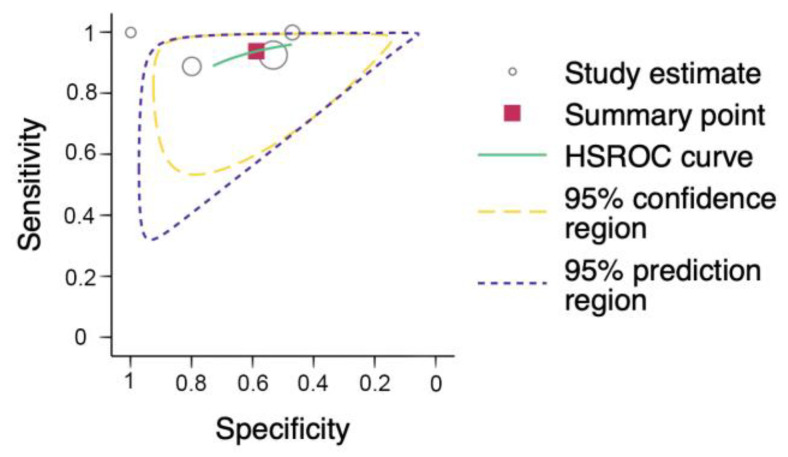
Meta-analysis with hierarchical summary receiver-operating characteristic (HSROC) curve.

After meta analysis by Stata 18 metandi command [22], the sensitivity of EP-FCU to diagnose diastolic dysfunction was 94% (95% CI: 87 to 97%) and the specificity was 59% (95% CI: 42 to 74%), with a summary PLR of 2.27 (95% CI: 1.55 to 3.33) and a summary NLR of 0.11 (95% CI: 0.05 to 0.21), supporting EP-FCU as a rule-out tool based on the results of cardiologist-performed TTE (Figure 3 and Table 3).

We performed a sensitivity analysis excluding the small—sample study by Moeller et al. (2019; *n* = 20) [25] using Meta-DiSc 2.0 [24]. The pooled estimates were sensitivity 93% (95% CI: 87% to 96%), specificity 59% (95% CI: 42% to 75%) and PLR 2.27 (95% CI: 1.36 to 3.18), NLR 0.12 (95% CI: 0.05 to 0.19). Absolute differences were 1 percentage point for sensitivity and 0.01 for NLR, with broadly overlapping CIs, indicating robustness of our pooled estimates after exclusion of that study [25].

### 3.4. Clinical Utility of EP-FCU to Diagnose Diastolic Dysfunction

**Figure 4:**
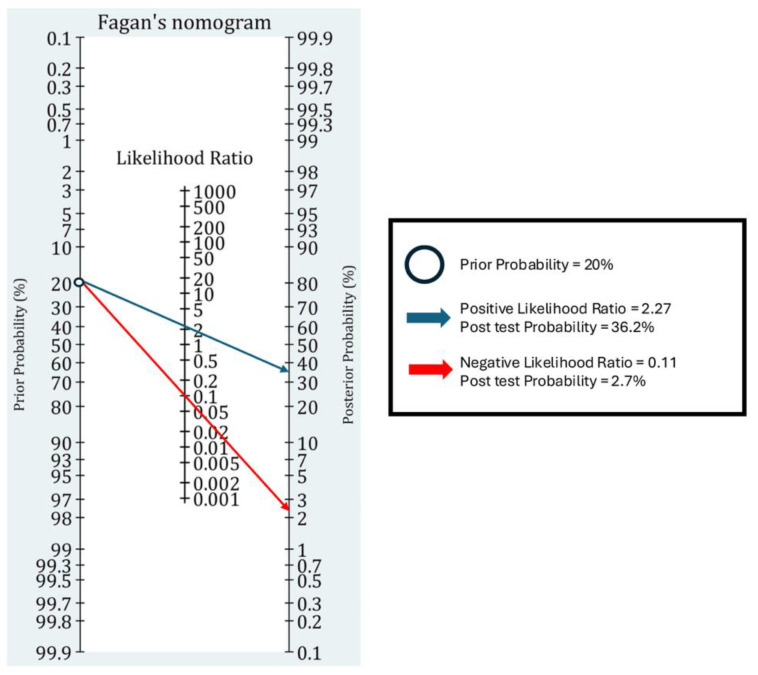
Fagan nomogram illustrating the 20% pre-test probability scenario, showing the association of a positive EP-FCU result (blue arrow) with a post-test probability of ~36.2% using positive likelihood ratio 2.27, and that of a negative result (red arrow) with a post-test probability of ~2.7% based on a negative likelihood ratio of 0.11.

The clinical utility of EP-FCU was evaluated using a scenario-based approach. We assumed pre-test probabilities of 10%, 20%, and 30% to reflect plausible ranges in the ED populations. Post-test probabilities for positive and negative EP-FCU were derived from the meta-analytic positive and negative likelihood ratios using odds-based Bayes equations. The 20% scenario was displayed in a Fagan nomogram for demonstration (Figure 4) with all scenarios being summarized numerically in Table 4.

Using the summary estimates (PLR = 2.27; NLR = 0.11) in the main analysis, the post-test probabilities after a positive EP-FCU result were 20.1%, 36.2%, and 49.3% for pre-test probabilities of 10%, 20%, and 30%, respectively; after a negative result, they were 1.2%, 2.7%, and 4.5%, respectively (Table 4).

For decision-making, we used a 2% rule—out threshold for ED safety considerations as previously described [27]. Based on our calculated NLR of 0.11, the maximal pre-test probability to achieve a 2% rule-out threshold after a negative result was 16%; only the 10% scenario met this threshold. These estimates support a triage role for EP-FCU in ruling out DD in lower-prevalence settings. In contrast, in higher pre-test contexts, our results indicated the need for additional diagnostic parameters (e.g., natriuretic peptides) to rule out the diagnosis. Our findings also showed that positive EP-FCU results increased the post-test probability of DD to 20–49% across different scenarios (Table 4), which is insufficient for ruling in DD and would warrant confirmatory testing (e.g., formal TTE).

### 3.5. Assessment of Methodological Quality

**Figure 5:**
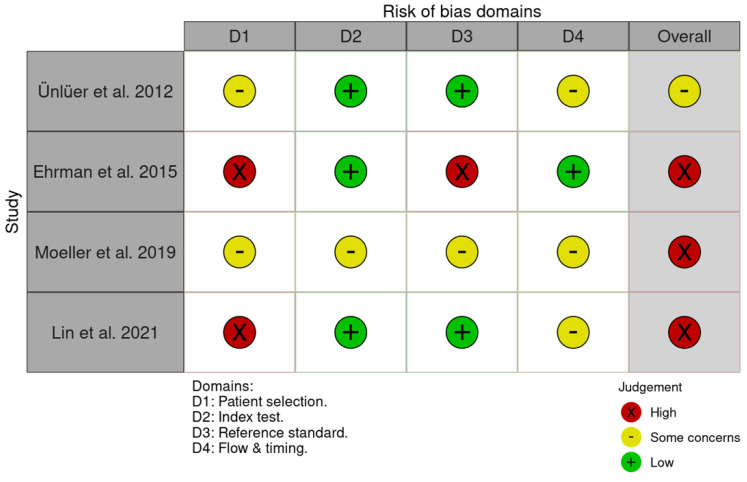
Risk of bias assessment of the included studies. Data were extracted from studies by Ünlüer et al., 2012 [10]; Ehrman et al., 2015 [11]; Moeller et al., 2019 [25], and Lin et al., 2021 [12].

The QUADAS-2 tool was used to assess the risk of bias across the four studies, with the results presented in a traffic-light plot generated by the Robvis tool [22] (Figure 5).

In the patient-selection domain, the studies by Ehrman et al. (2015) [11] and Lin et al. (2021) [12] were rated as having a high risk due to convenience sampling. In the reference-standard domain, the study by Ehrman et al. (2015) [11] presented a risk of incorporation bias, as the cardiologist-reviewed emergency physician-acquired echo clips were directly used to classify diastolic function. This approach may lead to incorporation and misclassification biases, particularly if scanning techniques were suboptimal or key measurements [e.g., left atrial volume and peak tricuspid regurgitation (TR) velocity] routinely included in formal TTE were omitted. In flow-and-timing domain, the studies by Ünlüer et al. (2012) [10] and Lin et al. (2021) [12] were judged to present with an unclear risk because the interval between EP-FCU and formal TTE varied from less than two hours to up to eight hours, during which patient hemodynamics might change, posing a risk of misclassification bias. Currently, no consensus exists regarding the optimal interval between these imaging assessments within which the hemodynamic fluctuation is minimal to ensure optimal data comparison. Finally, Moeller et al. (2019) [25] provided insufficient data to answer signaling questions across all four domains; thus, the risk of bias for all four domains was unclear, resulting in an overall high risk of bias for their study.

## 4. Discussion

### 4.1. Key Findings

To the best of our knowledge, the current study is the first systematic review and meta-analysis to evaluate EP-FCU accuracy in identifying diastolic dysfunction in patients with suspected acute heart failure. Our analysis demonstrated that EP-FCU achieved high sensitivity (94%, 95% CI: 87 to 97%) and a low NLR (0.11, 95% CI: 0.05 to 0.21) but moderate specificity (59%, 95% CI: 42 to 74%) and a modest PLR (2.27, 95% CI: 1.55 to 3.33), supporting greater utility in ruling out rather than ruling in diastolic dysfunction in patients with suspected acute heart failure.

In scenario-based clinical-utility analyses (pre-test 10%/20%/30%), a negative EP-FCU reduced post-test risk to 1.2%, 2.7%, and 4.5%, respectively; under a 2% rule-out threshold relevant to ED practice, this supports a triage role for rule out DD primarily in lower-prevalence settings (≤16% pre-test). Accordingly, because at higher pre-test probabilities (~20–30%) even a negative EP-FCU leaves post-test risks >2% (~2.7–4.5%) and a positive EP-FCU alone yields only ~20–49%—insufficient to rule-in—formal TTE (and/or a structured diagnostic pathway) is recommended when pre-test risk exceeds 16% or when EP-FCU is positive/indeterminate or image quality is limited.

### 4.2. Integration with International Guidelines

The findings of this review are notably relevant in clinical practice, as they highlight an evolving tension between current consensus-driven clinical recommendations and recent shifts in training paradigms within emergency medicine. On the one hand, foundational consensus statements from the ASE/ACEP (2010) [15] and ESC/EACVI (2013) [16] have traditionally emphasized the inappropriateness of using FCU without comprehensive training for a definitive assessment of diastolic function. On the other hand, the recent 2023 AEMUS Core Content revision explicitly lists diastolic function assessment as a core competency [17], signaling a significant shift in training expectations.

Our systematic review may provide useful evidence to navigate this transitional period. In contrast with the cautious stance of earlier guidelines that denied the reliability of EP-FCU as a diagnostic modality for diastolic dysfunction [15,16], the excellent sensitivity (94%) and low NLR (0.11) identified in the current study supported its use as a triage tool for ruling out this condition. Our findings are in line with the recommendation of the recent AEMUS guidelines that included ultrasound skills in the core training program for emergency physicians [17]. Nevertheless, the moderate specificity (59%) and modest PLR (2.27) rendered it inappropriate as a standalone tool for ruling in the diagnosis. In this way, our work may help bridge the gap between these perspectives by defining both the current utility and the clear limitations of EP-FCU.

### 4.3. Limitations

Consistent with GRADE for test accuracy [18,28], we summarize limitations across four domains—external validity (applicability), internal validity (risk of bias), inconsistency (heterogeneity), and imprecision—and then add a brief note on natriuretic peptides for clinical context.

External validity (applicability) of our study result for routine ED practice may be limited by operator expertise. Therefore, the involvement of fellowship-trained physicians in three of the four included studies [11,12,25] may not reflect the average ED setting. Applicability is also constrained by the participant spectrum. Two studies (Ünlüer et al. [10], Lin et al. [12]) excluding common comorbidities such as atrial fibrillation, significant valvular disease, or tachycardia—conditions frequently encountered in undifferentiated ED presentations—so generalizability to real world ED populations may be limited.

The internal validity of the included studies may be threatened by several risks of bias, which was evaluated by QUADAS-2. In the patient selection domain, the studies by Ehrman et al. (2015) [11] and Lin et al. (2021) [12] use convenience sampling rather than consecutive/random sampling, which “may produce estimates of test accuracy that do not reflect the performance of the test in clinical practice”. Two studies (Ünlüer et al. [10], Lin et al. [12]) also excluded common comorbidities (atrial fibrillation, significant valvular disease, tachycardia), risking patient-selection (spectrum) bias—in addition to the applicability concerns noted above. Also the recruitment of patients with both preserved and reduced ejection fraction in the studies by Ehrman et al. [11] and Lin et al. [12] may have contributed spectrum bias, as diastolic parameters such as E/A ratio and E/e′ behave differently across the EF spectrum. Reference standard-related bias was present in three out of the four studies. In the study by Ehrman et al., cardiologists reinterpreted the images acquired by emergency physicians, thereby introducing incorporation bias and misclassification bias. In addition, the timing between EP-FCU and formal TTE varied significantly; it was less than two hours in Ünlüer et al. [10], but up to eight hours in that by Lin et al. [12]. Such time gaps could allow for changes in hemodynamic status, potentially leading to misclassification bias.

The inconsistency (heterogeneity) in our findings may be explained by the wide variation in index test protocol across our included studies, in which EP-FCU did not represent a single uniform index test but rather a spectrum different protocols—from transmitral inflow only (Ünlüer) [10] to protocols incorporating TDI and left atrial size (Ehrman [11]; Lin [12]). Such a discrepancy in protocols implied the utilization of different positivity thresholds, thereby producing a threshold effect—movement along the ROC curve with higher sensitivity at the expense of specificity, and vice versa. This phenomenon is visible in the coupled forest plots (Figure 2). Specifically, setting aside the small-sample-size study by Moeller et al. [25], the other studies formed a V-shaped arrangement consistent with the presence of a threshold effect. The wide variation in EP-FCU index test protocols across our included studies may be a significant limitation of our study outcome. Further large-scale clinical investigations focusing on specific protocols are warranted to verify our findings.

Imprecision of our study outcomes was also notable. The inclusion of only four studies (327 enrolled; 298 analyzed) in our meta-analysis contributed to a wide confidence region of the summary sensitivity and specificity.

Lastly, all four studies based their diagnosis of DD only on echocardiography. On the other hand, although a number of heart failure diagnostic guidelines recommend incorporating natriuretic peptides (BNP/NT-proBNP) into the diagnostic pathways [2,4,5,6], prior studies indicated that up to about 30% of patients with HFpEF may have normal BNP values despite the presence of symptoms, echocardiographic abnormalities, and evidence derived from invasive hemodynamic procedures (e.g., elevated filling pressures) [5,29,30].

### 4.4. Future Research Directions

Our findings, which suggested the applicability of EP-FCU as a rule out triage tool for undifferentiated dyspnea in low prevalence ED settings, warranted validation from further large-scale studies that should enroll average emergency physicians without ultrasound fellowship training to better reflect the real-world ED scenario. Moreover, instead of convenience sampling of ED patients adopted in two of our included studies [11,12], consecutive or randomized sampling may help in minimizing potential sampling bias [18]. In addition, common comorbidities such as atrial fibrillation, significant valvular disease, and tachycardia may not be routinely excluded to avoid spectrum bias [18]. Furthermore, the observed protocol variation indicates that future EP-FCU should emphasize the utilization of a fixed positivity threshold (e.g., ASE guidelines-aligned) coupled with the adoption of a standardized protocol (e.g., measurement parameters, interval between EP-FCU and formal TTE) as well as the implementation of a standardized EP-FCU training program and imaging quality control measures to minimize heterogeneity through reducing the threshold effect and decreasing equipment and operator variability. Finally, the development and validation of artificial intelligence (AI)-assisted quantification tools with a strong emphasis on applicability in the evaluation of cardiac function in an emergency medical setting may further broaden the horizon in this area [31].

## 5. Conclusions

To our best knowledge, this is the first systematic review and meta-analysis to evaluate the diagnostic accuracy of EP-FCU for assessing diastolic dysfunction in patients with suspected acute heart failure. Our findings demonstrated that while EP-FCU showed excellent sensitivity (94%, 95% CI: 87 to 97%) and a low NLR (0.11, 95% CI: 0.05 to 0.21) that supported its use as an effective tool to rule out diastolic dysfunction, its moderate specificity (59%, 95% CI: 42 to 74%) and limited PLR (2.27, 95% CI: 1.55 to 3.33) precluded its standalone use as a definitive diagnostic modality. Our results highlighted the viability of EP-FCU as an effective initial point-of-care triage tool in the differential diagnosis of patients presenting with acute dyspnea based on comparison with formal TTE that still serves as the standard for definitive diagnosis as recommended by the ASE/ACEP (2010) and ESC/EACVI (2013) guidelines [15,16].

## Figures and Tables

**Table 1:** Baseline characteristics of included studies.

Study (Year)	Sample Size (*n*)	Male (%)	Age ± SD (Years)	Definition of DD by EP
Ünlüer et al. (2012) [10]	69			Grade I is an abnormal relaxation pattern characterized by a prolonged IVRT, decreased E-wave amplitude, prolonged deceleration time, increased A-wave amplitude, and a ratio of early diastolic (E) to peak late diastolic (A) velocities, E/A, of less than 1
		NR	63 ± 9	2. Grade II is a pseudonormal pattern characterized by normalization of transmitral flow E/A ratio
				3. Grade III is a restrictive pattern characterized by shortened IVRT, increased E-wave amplitude, decreased deceleration time and a ratio of E/A greater than 2
Ehrman et al. (2015) [11]				Diastolic dysfunction graded by simplified 2009 ASE criteria using Doppler and TDI:
	62	48%	56 ± 14	Normal: E’sept ≥ 8 and E’lat ≥ 10
				2. Grade 1: E’sept < 8 or E’lat < 10 and E/E’avg < 8 or E/A < 0.8 3. Grade 2: E’sept < 8 or E’lat < 10 and E/E’avg 8–12 or E/A 0.8–1.5 4. Grade 3: E’sept < 8 or E’lat < 10 and E/E’avg > 12 or E/A > 1.5
Moeller et al. 2019 [25]	20	NR	NR	NR
Lin et al. (2021) [12]				Normal: E > A and >50% of other specified diastolic variables are normal
	176	65%	64 ± 14	2. Mild (Grade 1): E ≤ A (sole criterion for diagnosis)
				3. Moderate (Grade 2): E > A (E/A ratio < 2:1) + ≥1 abnormal TDI * measurement 4. Severe (Grade 3): E > A (E/A ratio ≥ 2:1) + ≥1 abnormal TDI measurement + LA diameter > 4 cm

DD = diastolic dysfunction; EP = emergency physician; IVRT = isovolumic relaxation time; LA = left atrium; NR = not reported; SD = standard deviation; TDI = Tissue Doppler imaging. * TDI measurements in the study by Lin et al. including septal e′, lateral e′, E/e′ ratios, and LA diameter [11].

**Table 2:** Diagnostic performance of emergency physician-performed focused cardiac ultrasound for diastolic dysfunction compared with reference standards.

Study (Year)	Sensitivity (95% CI)	Specificity (95% CI)	PLR	NLR
Ünlüer et al. (2012) [10]	89% (77 to 96)	80% (52 to 96)	4.44 (1.6 to 12.28)	0.14 (0.06 to 0.31)
Ehrman et al. (2015) [11]	100% (87 to 100)	47% (23 to 72)	1.89 (1.21 to 2.96)	0 **
Moeller et al. (2019) [25]	100% (82 to 100)	100% (2.5 to 100)	not estimable *	0 **
Lin et al. (2021) [12]	93% (87 to 96)	53% (34 to 72)	1.98 (1.35 to 2.92)	0.14 (0.07 to 0.28)

PLR = positive likelihood ratio; NLR = negative likelihood ratio; * PLR not estimable due to zero false positives; ** NLR = 0 due to zero false negatives.

**Table 3:** Summary points for diagnostic accuracy.

Summary Point	Estimate	95% CI
sensitivity	94%	87% to 97%
specificity	59%	42% to 74%
PLR	2.27	1.55 to 3.33
NLR	0.11	0.05 to 0.21

CI = confidence interval; PLR = positive likelihood ratio; NLR = negative likelihood ratio.

**Table 4:** Post-test probabilities across pre-test scenarios.

**Pre-Test Probability**	**PLR = 2.27**	**NLR = 0.11**
10%	20.1%	1.2%
20%	36.2%	2.7%
30%	49.3%	4.5%

PLR = positive likelihood ratio; NLR = negative likelihood ratio.

## Data Availability

Data sharing is not applicable.

## Supplementary Materials

The following supporting information can be downloaded at https://www.mdpi.com/article/10.3390/jcm14217726/s1, Supplementary File S1: PRISMA-DTA checklist; Supplementary File S2: PRISMA-DTA for abstract checklist; Supplementary File S3: keywords and search strategy; Supplementary File S4: Studies excluded and reasons for exclusion following full-text review.

## Abbreviations

The following abbreviations are used in this manuscript:

ABEM	American Board of Emergency Medicine
ACEP	American College of Emergency Physicians
AEMUS	Advanced Emergency Medicine Ultrasonography
AI	Artificial Intelligence
ASE	American Society of Echocardiography
CENTRAL	Cochrane Central Register of Controlled Trials
CI	Confidence Interval
DD	Diastolic Dysfunction
ED	Emergency Department
EF	Ejection Fraction
EACVI	European Association of Cardiovascular Imaging
EP	Emergency Physician
EP-FCU	Emergency Physician-performed Focused Cardiac Ultrasound
ESC	European Society of Cardiology
FCU	Focused Cardiac Ultrasound
HF	Heart Failure
HFpEF	Heart Failure with preserved Ejection Fraction
HFrEF	Heart Failure with reduced Ejection Fraction
HSROC	Hierarchical Summary Receiver-Operating Characteristic
LA	Left Atrium
MeSH	Medical Subject Headings
NLR	Negative Likelihood Ratio
PLR	Positive Likelihood Ratio
PRISMA-DTA	Preferred Reporting Items for Systematic Reviews and Meta-Analyses of Diagnostic Test Accuracy
PROSPERO	International Prospective Register of Systematic Reviews
QUADAS-2	Quality Assessment of Diagnostic Accuracy Studies—2
Rayyan	Web-based screening tool for systematic reviews
RevMan	Review Manager
TDI	Tissue Doppler Imaging
TR	Tricuspid Regurgitation
TTE	Transthoracic Echocardiography
