# Supplementary material for: Accuracy of Emergency Physician-Performed Echocardiography for Diastolic Dysfunction in Suspected Acute Heart Failure: A Systematic Review and Meta-Analysis
Source: J Clin Med. 2025 Oct 30;14(21):7726. doi: 10.3390/jcm14217726 (PMC12608506; doi:10.3390/jcm14217726)
Supplement: Supplementary file 1 [file jcm-14-07726-s001.zip › Supplementary File S2, PRISMA-DTA for abstract checklist.pdf]

PRISMA-DTA for Abstracts Checklist

Section/topic	#	PRISMA-DTA for Abstracts Checklist item	Reported on page #
**TITLE and PURPOSE**			
Title	1	Identify the report as a systematic review (+/- meta-analysis) of diagnostic test accuracy (DTA) studies.	1
Objectives	2	Indicate the research question, including components such as participants, index test, and target conditions.	1
**METHODS**			
Eligibility criteria	3	Include study characteristics used as criteria for eligibility.	1
Information sources	4	List the key databases searched and the search dates.	1
Risk of bias & applicability	5	Indicate the methods of assessing risk of bias and applicability.	1
Synthesis of results	A1	Indicate the methods for the data synthesis.	1
**RESULTS**			
Included studies	6	Indicate the number and type of included studies and the participants and relevant characteristics of the studies (including the reference standard).	1
Synthesis of results	7	Include the results for the analysis of diagnostic accuracy, preferably indicating the number of studies and participants. Describe test accuracy including variability; if meta-analysis was done, include summary results and confidence intervals.	1
**DISCUSSION**			
Strengths and limitations	9	Provide a brief summary of the strengths and limitations of the evidence	1
Interpretation	10	Provide a general interpretation of the results and the important implications.	1
**OTHER**			
Funding	11	Indicate the primary source of funding for the review.	1
Registration	12	Provide the registration number and the registry name	1

Adapted From: McInnes MDF, Moher D, Thombs BD, McGrath TA, Bossuyt PM, The PRISMA-DTA Group (2018). Preferred Reporting Items for a Systematic Review and Meta-analysis of Diagnostic Test Accuracy Studies: The PRISMA-DTA Statement. JAMA. 2018 Jan 23;319(4):388-396. doi: 10.1001/jama.2017.19163.
