# Supplementary material for: Accuracy of Emergency Physician-Performed Echocardiography for Diastolic Dysfunction in Suspected Acute Heart Failure: A Systematic Review and Meta-Analysis
Source: J Clin Med. 2025 Oct 30;14(21):7726. doi: 10.3390/jcm14217726 (PMC12608506; doi:10.3390/jcm14217726)
Supplement: Supplementary file 1 [file jcm-14-07726-s001.zip › Supplementary File S3, keywords and search strategy.pdf]

Supplementary File S3: keywords and search strategy

pubmed	(("Heart Failure, Diastolic"[Mesh] OR "diastolic dysfunction" OR "diastolic function" OR "diastolic heart failure") AND (echocardiography OR ultrasound OR Doppler OR "echocardiography, doppler"[MeSH]) AND ("Emergency Medicine"[Mesh] OR "Emergency Service, Hospital"[Mesh] OR "emergency physician" OR "Emergency department"))
embase	('heart failure, diastolic'/exp OR 'heart failure, diastolic' OR 'diastolic dysfunction'/exp OR 'diastolic dysfunction' OR 'diastolic function'/exp OR 'diastolic function' OR 'diastolic heart failure'/exp OR 'diastolic heart failure') AND ('echocardiography'/exp OR 'echocardiography' OR 'echocardiography, doppler'/exp OR 'echocardiography, doppler' OR echo* OR doppl* OR 'ultrasound'/exp OR ultrasound) AND ('emergency medicine'/exp OR 'emergency medicine' OR 'emergency ward'/exp OR 'emergency ward' OR 'emergency

	physician'/exp OR 'emergency physician' OR 'emergency department'/exp OR 'emergency department')
cochrane	("diastolic dysfunction" OR "diastolic heart failure" OR "diastolic function") AND (echocardiography OR ultrasound OR doppler) AND ("emergency medicine" OR "emergency department" OR "emergency physician")
