# Supplementary material for: Accuracy of Emergency Physician-Performed Echocardiography for Diastolic Dysfunction in Suspected Acute Heart Failure: A Systematic Review and Meta-Analysis
Source: J Clin Med. 2025 Oct 30;14(21):7726. doi: 10.3390/jcm14217726 (PMC12608506; doi:10.3390/jcm14217726)
Supplement: Supplementary file 1 [file jcm-14-07726-s001.zip › Supplementary File S4, Full text screen studies excluded in the systematic review.pdf]

Supplementary File S4: Studies excluded in the current systematic review

---

1. Simulation study / no real patient involved

1. Adhikari S. Can emergency physicians accurately identify complex abnormalities on point-of-care echocardiogram? *Annals of Emergency Medicine*. 2013;62(4):S78.
   Detailed reason for exclusion: evaluated clinicians' diagnostic accuracy according to pre-recorded images or scenarios rather than performance on live patients in a clinical setting
   DOI: 10.1016/j.annemergmed.2013.07.034
2. Kluger SB. Ability of emergency medicine residents in the diagnosis of CHF with a preserved ejection fraction by echocardiogram. *American Journal of Emergency Medicine*. 2018;36(6):1113–1114.
   Detailed reason for exclusion: evaluated clinicians' diagnostic accuracy according to pre-recorded images or scenarios rather than performance on live patients in a clinical setting
   DOI: 10.1016/j.ajem.2017.10.033

---

2. Outcome not relevant

1. Arnone MI. Assessment of E/A ratio helps emergency clinicians in the management of patients with acute dyspnea. *Internal and Emergency Medicine*. 2023;18(6):1823–1830.
   DOI: 10.1007/s11739-023-03279-8
2. Park CH. A pilot study evaluating LV diastolic function with M-mode measurement of mitral valve movement in the parasternal long axis view. *Diagnostics*. 2023;13(14):2412.
   DOI: 10.3390/diagnostics13142412
3. Saul T. The inter-rater reliability of echocardiographic diastolic function evaluation among emergency physician sonographers. *Journal of Emergency Medicine*. 2016;51(4):411–417.
   DOI: 10.1016/j.jemermed.2016.06.031

---

3. Patient population mismatch

1. Colla JS. Identification of subclinical cardiac dysfunction using bedside echocardiograms on emergency department patients by emergency physicians. *Annals of Emergency Medicine*. 2014;64(4):S121.
   Detailed reason for exclusion: enrolled asymptomatic patients with elevated blood pressure to investigate subclinical cardiac dysfunction
   DOI: 10.1016/j.annemergmed.2014.07.373
2. Del Rios M. Emergency physician use of tissue Doppler bedside echocardiography in detecting diastolic dysfunction: an exploratory study. *Critical Ultrasound Journal*. 2018;10(1):5.
   Detailed reason for exclusion: enrolled asymptomatic patients with elevated blood pressure to investigate subclinical cardiac dysfunction
   DOI: 10.1186/s13089-018-0084-5
3. Gottlieb M. Diagnostic accuracy of artificial intelligence for identifying systolic and diastolic cardiac dysfunction in the emergency department. *American Journal of Emergency Medicine*. 2024;86:115–119.
   DOI: 10.1016/j.ajem.2024.10.019
4. Kotini-Shah P. A simplified approach to screen for diastolic dysfunction using limited bedside echo by emergency physicians. *Academic Emergency Medicine*. 2015;22(5):S379.
   Detailed reason for exclusion: explicitly recruited patients without clinical signs of heart failure
   DOI: 10.1111/acem.12644

---

4. Review article

1. Chenkin J. Contemporary application of point-of-care echocardiography in the emergency department. *Canadian Journal of Cardiology*. 2018;34(2):109–116.
   DOI: 10.1016/j.cjca.2017.08.018
2. Choi W. Role of point-of-care ultrasound in critical care and emergency medicine: update and future perspective. *Clinical and Experimental Emergency Medicine*. 2023;10(4):363–381.
   DOI: 10.15441/ceem.23.101
3. Dhont S. Non-invasive imaging in acute decompensated heart failure with preserved ejection fraction. *European Heart Journal: Acute Cardiovascular Care*. 2024;13(7):575–582.
   DOI: 10.1093/ehjacc/zuae041
4. Holst JM. Heart failure with preserved ejection fraction: echocardiographic VALVE protocol for emergency physicians. *European Journal of Emergency Medicine*. 2014;21(6):394–402.
   DOI: 10.1097/MEJ.0000000000000093

5. Via G. Diagnosis of diastolic dysfunction in the emergency department: really at reach for minimally trained sonologists? A call for a wise approach to heart failure with preserved ejection fraction diagnosis in the ER. *Critical Ultrasound Journal*. 2018;10(1)
   DOI: 10.1186/s13089-018-0107-2
6. Weekes AJ. Emergency echocardiography. *Emergency Medicine Clinics of North America*. 2011;29(4):759–787.
   DOI: 10.1016/j.emc.2011.08.002

---

5. Different index test

1. Filipiak D. A novel handheld ultrasound device equipped with spectral Doppler—efficacy of left ventricle diastolic function parameters assessment. *European Heart Journal – Cardiovascular Imaging*. 2025;26:i639–i640.
   DOI: 10.1093/ehjci/jeae333.416
